# 基于二维材料的色谱固定相制备及应用

**DOI:** 10.3724/SP.J.1123.2024.01022

**Published:** 2024-06-08

**Authors:** Desheng ZHENG, Wenqi TANG, Jianping ZHU, Zhiyuan GU

**Affiliations:** 南京师范大学化学与材料科学学院, 江苏 南京 210023; School of Chemistry and Materials Science, Nanjing Normal University, Nanjing 210023, China

**Keywords:** 二维材料, 金属有机框架, 共价有机框架, 色谱固定相, 综述, two-dimensional materials, metal organic frameworks (MOFs), covalent organic frameworks (COFs), chromatographic stationary phase, review

## Abstract

自2004年首次报道以来,二维材料因具有纳米级厚度、高比表面积、独特的层状结构和优异的机械性能等特点,受到了广泛关注。在导电、导热、气体存储和分离、膜分离以及催化等领域,二维材料均展现出了广阔的应用前景;其中,最为知名的二维材料是石墨烯,其是由单层碳原子构成的二维晶格结构,拥有较大的比表面积和优良的吸附性能,因而备受青睐。除此之外,二维材料还包括类石墨相氮化碳、氮化硼、过渡金属硫化物等。随着网状化学的不断发展,二维多孔框架材料逐渐受到关注,这类材料拥有纳米片状的形态、一维的孔道结构以及特殊的层间作用力,具有作用力丰富和可设计性强等优势,在色谱分离领域展现出了巨大的应用潜力。本文首先简要介绍了二维材料的合成方法与分离机理,随后着重介绍了近年来二维多孔框架材料在气相色谱及液相色谱固定相应用方面的最新研究进展。同时,本文也涉及了一些其他二维材料在色谱领域的应用,并对二维材料在色谱分离领域的发展前景进行了展望,期望为基于二维材料的色谱分离材料设计及应用提供参考。

## Abstract (en)

The stationary phase is the heart of chromatographic separation technology and a critical contributor to the overall separation performance of a chromatographic separation technique. However, traditional silicon-based materials designed for this purpose usually feature complex preparation processes, suboptimal permeability, pronounced mass-transfer resistance, and limited pH-range compatibility. These limitations have spurred ongoing research efforts aimed at developing new chromatographic stationary phases characterized by higher separation efficiency, adaptable selectivity, and a broader scope of applicability. In this context, the scientific community has made significant strides toward the development of new-generation materials suitable for use as chromatographic stationary phases. These materials include carbon-based nanomaterial arrays, carbon quantum dots, and two-dimensional (2D) materials. 2D-materials are characterized by nanometer-scale thicknesses, extensive specific surface areas, distinctive layered structures, and outstanding mechanical properties under standard conditions. Thus, these materials demonstrate excellent utility in various applications, such as electrical and thermal conductivity enhancements, gas storage and separation solutions, membrane separation technologies, and catalysis. Graphene, which is arguably the most popular 2D-material used for chromatographic separation, consists of a 2D-lattice of carbon atoms arranged in a single layer, with a large specific surface area and efficient adsorption properties. Its widespread adoption in research and various industries is a testament to its versatility and effectiveness. In addition to graphene, the scientific community has developed various 2D-materials that mirror the layered structures of graphene, such as boron nitride, transition-metal sulfides, and 2D porous organic frameworks, all of which offer unique advantages. 2D porous organic frameworks, in particular, have received attention because of their nanosheet morphology, one-dimensional pores, and special interlayer forces; thus, these frameworks are considered promising candidate chromatographic stationary phase materials. Such recognition is especially true for 2D-metal organic frameworks (MOFs) and 2D-covalent organic frameworks (COFs), which exhibit low densities, high porosities, and substantial specific surface areas. The modifiability of these materials, in terms of pore size, shape, functional groups, and layer-stacking arrangements allows for excellent separation selectivity, highlighting their promising potential in chromatographic separation. Compared with their three-dimensional counterparts, 2D-MOFs feature a simple pore structure that offers reduced mass-transfer resistance and enhanced column efficiency. These attributes highlight the advantages of 2D-MOF nanosheets as chromatographic stationary phases. Similarly, 2D-COFs, given their high specific surface area and porosity, not only exhibit great thermal stability and chemical tolerance but also support a wide selection of solvents and operational conditions. Therefore, their role in the preparation of chromatographic stationary phases is considered highly promising. This review discusses the latest research developments in 2D porous organic framework materials in the context of gas- and liquid-chromatographic stationary phases. It introduces the synthesis methods for these novel materials, elucidates their retention mechanisms, and describes the applications of other 2D-materials, such as graphene, its derivatives, graphitic carbon nitride, and boron nitride, in chromatography. This review aims to shed light on the promising development prospects and future directions of 2D-materials in the field of chromatographic separation, offering valuable insights into the rational design and application of new 2D-materials in chromatography.

色谱是一种针对复杂样品组分定性、定量检测的重要分离分析技术,其利用分析物与固定相之间的相互作用来实现复杂体系的分离分析,已被广泛应用于环境监测^[1]^、生命科学^[2,3]^、食品安全监测^[4]^和药物分析^[5]^等领域。作为色谱分离技术的核心,色谱固定相对分离性能有着决定性作用。色谱技术的进步在很大程度上取决于高分离效率的新型固定相的开发设计,以能够满足对各种分析物的高选择性和高灵敏度检测需求。传统的硅基色谱固定相材料存在制备工艺复杂、渗透性差、传质阻力高、pH范围窄等明显缺点^[6⇓-8]^,因此开发具有分离效率高、选择性灵活和适用性广泛的新型色谱固定相一直是分离分析科学领域的研究重点之一。近些年来,许多适用于色谱固定相的新型材料被开发出来,例如碳质纳米材料^[9]^、碳量子点^[10]^、二维材料^[11⇓⇓-14]^等。

2004年,Novoselov等^[15]^成功分离出单原子层的石墨材料——石墨烯(graphene),二维材料随之被提出。二维材料是由单层或几层原子组成的材料,其在至少一个方向上具有纳米级厚度,在其他两个方向上具有宏观尺寸。二维材料通常表现出堆叠的层状结构,层内原子间通过共价键连接,层与层之间通过范德华力进行堆叠。二维材料通常具有高比表面积、独特的电子结构以及高机械性能等特点,其在导电、导热、气体存储和分离、吸附、膜分离以及催化等领域均具有广阔的应用前景^[16]^。除石墨烯外,二维材料还包括类石墨相氮化碳^[17]^、氮化硼^[18]^、过渡金属硫化物^[19]^等。随着网状化学的发展^[20,21]^,通过特殊合成手段能够使多孔框架材料仅在平面内形成共价键连接的层状框架结构,而层与层之间通过弱相互作用力(如范德华力)进行堆叠,这种多孔框架材料具备了二维材料的特点,表现出二维纳米片形貌,也被称作二维多孔框架材料^[16,22⇓-24]^。二维多孔框架材料包括二维金属有机框架材料(2D-MOFs)和二维共价有机框架材料(2D-COFs),两种材料均具有一维孔道以及特殊的层间相互作用力,被证实具有作为色谱分离固定相的能力^[16]^。此外,二维多孔框架材料的孔径、形状以及层间堆叠排布方式都可以通过调控其构建单元来改变,从而实现不同的分离选择性^[25]^。

本文总结了基于二维材料的气相色谱和液相色谱固定相制备的研究进展,并重点介绍了二维多孔框架材料色谱固定相的制备及应用;同时,本文还介绍了部分其他二维材料色谱固定相的相关研究。最后,本文分析了二维材料色谱固定相发展所面临的难题,并提出了一些解决方案,旨在为高效二维材料色谱固定相的开发提供新思路。

## 1 二维材料的合成方法及色谱分离机理

从合成角度来看,涉及二维材料的合成过程可以归纳为两种策略,即自上而下策略和自下而上策略。自上而下策略通常需要预先制备出具有特殊结构的三维材料,这种三维材料由纳米级厚度的二维材料通过氢键或范德华力等弱相互作用力堆叠形成,能够为二维材料的自上而下剥离提供有利条件。通过施加其他外部作用力(如超声波、机械力等)于层间结构,可以破坏层间的弱作用力,从而实现二维材料的剥离。自上而下策略包括机械剥离、液相剥离、超声剥离、电化学剥离、离子交换剥离、插入剥离和化学剥离等。在自下而上策略中,二维材料是由原子或分子前驱体生成的,这些前驱体能够通过共价键、配位键等化学键相连接而生长成二维材料,或通过自组装生成结构复杂的二维材料。特别是对于二维多孔框架材料,自下而上策略要求在抑制晶体垂直生长的同时又不影响平面上两个维度方向的生长。自下而上策略包括界面合成、表面活性剂辅助合成、调控合成、拓扑化学转化合成等。与自上而下策略相比,自下而上策略能够实现二维材料的大规模生产。

基于二维材料的色谱固定相具有多种作用机制,包括空间位阻效应、氢键、偶极-偶极相互作用、*π-π*相互作用以及范德华力等,这些作用力会对不同分析物的分离产生不同影响。例如,偶极-偶极相互作用在卤代烷烃和卤代苯等极性化合物的分离过程中会产生强烈影响,而*π-π*相互作用对于芳香化合物的分离至关重要。深入理解以上作用机制将有助于色谱分离条件的优化,从而提高色谱分离效率和准确性。

## 2 二维材料用于气相色谱固定相

### 2.1 2D-MOFs

MOFs是一类由金属/金属簇与有机配体通过自组装形成的新型多孔材料,具有比表面积大、孔结构明确和可调性强等优点^[26]^,广泛应用在色谱分离领域^[27]^。2D-MOFs在MOFs的基础上,具有纳米级厚度,能够暴露出更多的活性位点,提供特殊的层间相互作用(如*π-π*相互作用)^[28]^。2D-MOFs的特殊结构有利于在分子水平上实现异构体(如烷烃异构体、苯系物等)的精准识别与分离^[16]^,其作为气相色谱固定相具有良好的应用前景。

Tao等^[16]^以*N*,*N*-二甲基甲酰胺(DMF)为溶剂、ZrCl_4_为金属中心、1,3,5-三(4-羧基苯基)苯(BTB)为配体、甲酸(FA)为抑制剂,通过溶剂热法合成了具有二维形貌的MOF(Zr-BTB-FA),见图1;其中,FA占据了Zr金属中心的上、下配位,抑制了MOF的轴向生长,从而使获得的Zr-BTB-FA呈现出二维片状形貌。Zr-BTB-FA之间的堆叠表现出了3种代表性的扭角,扭转角度分别为8°、14°和30°。此外,通过在乙醇中进行加热,单层Zr-BTB-FA纳米片之间会形成化学键Zr-O-Zr, Zr-BTB-FA的堆叠方式从原本具有一定角度的层间不规则堆叠转变为层间规则堆叠,并且纳米片层间产生了高度有序的亚纳米微孔。将规则堆叠的Zr-BTB-FA作为气相色谱固定相,能够实现6种异构体的分离,尤其对于对位异构体,Zr-BTB-FA展现出了良好的选择性。实验结果说明,MOF纳米片层之间的堆叠方式对MOF的性质和应用有着明显影响。随后,Tang等^[25]^利用Zr-BTB纳米片与甲苯和乙酸乙酯之间的主客体相互作用,得到了两种不同堆叠方式的2D-Zr-BTB纳米片(图2),其中两种纳米片的扭转角度分别为12°、18°、24°、6°以及18°、24°、30°。此外,该研究进一步发现,将长链烷烃作为客体分子垂直插入纳米片的孔道后,由于空间位阻和疏水相互作用,2D-Zr-BTB的堆叠方式转变为无扭转角度的规则堆叠,并对苯系异构体展现出了良好的分离能力。

**图 1:**
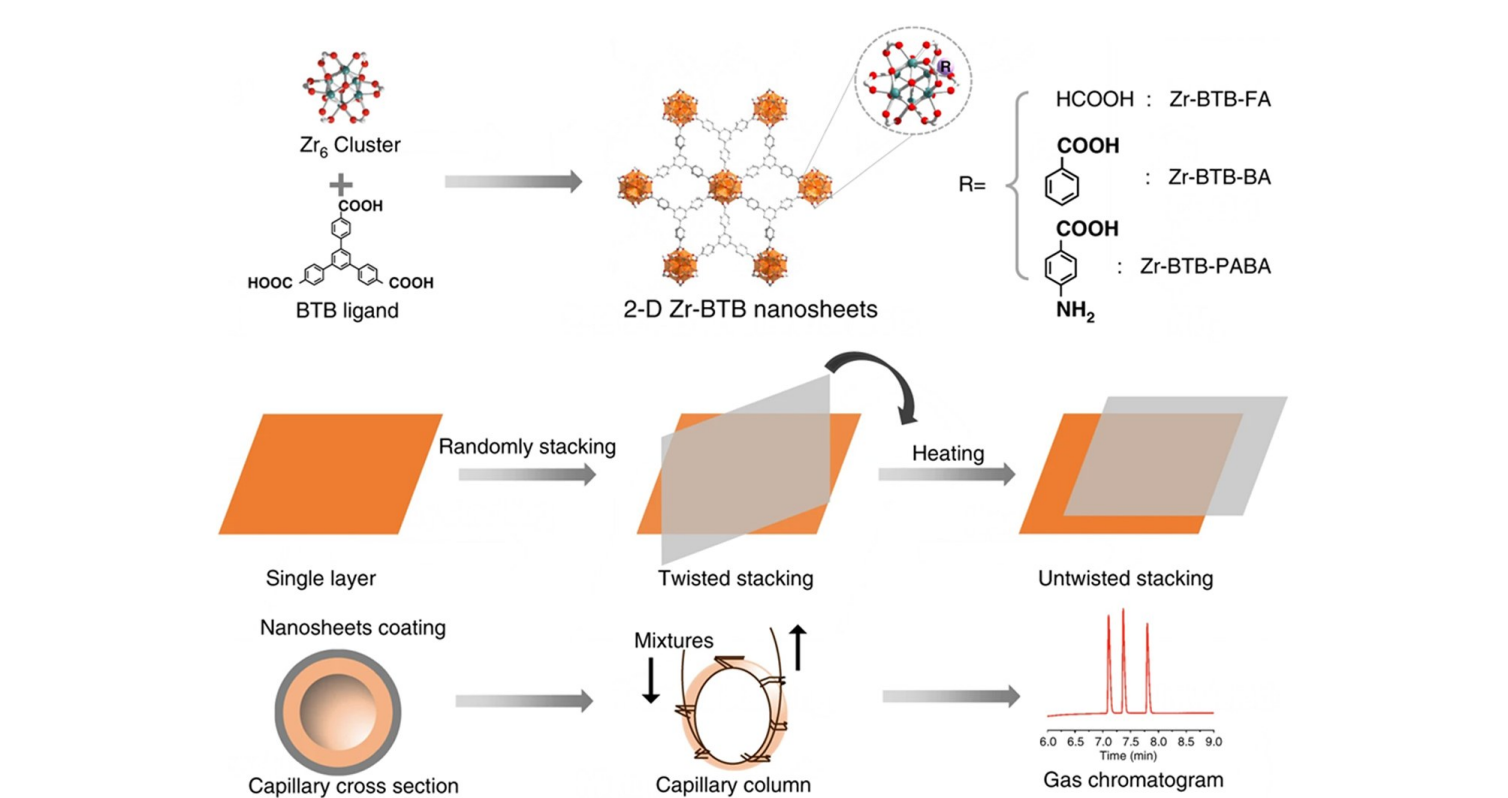
Zr-BTB-FA纳米片的合成过程及色谱分离性能^[16]^

**图 2:**
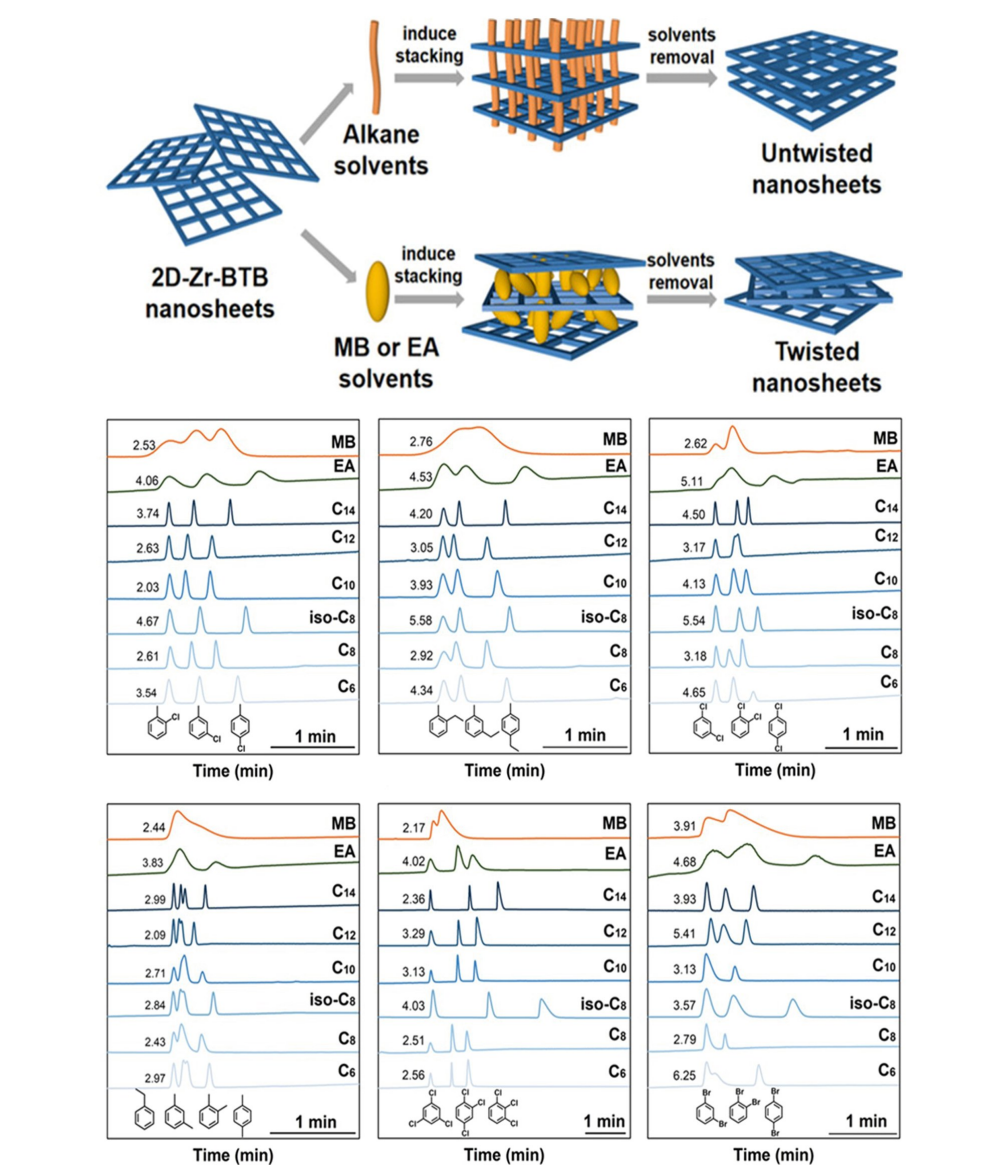
不同堆叠方式的2D-Zr-BTB制备流程及色谱分离性能^[25]^

最近,Tang等^[29]^进一步探究了不同链长客体分子对Zr-BTB堆叠模式的影响。该工作利用“客体扭矩扳手策略”,选择了一系列芳香化合物分子(包括甲苯(P-C_1_)、乙苯(P-C_2_)、丙苯(P-C_3_)和戊苯(P-C_5_))和酯类分子(包括乙酸乙酯(A-C_2_)、乙酸丙酯(A-C_3_)和乙酸丁酯(A-C_4_))作为扭矩扳手,以控制二维纳米片的层间堆积模式。根据主客体化学原理,通过改变“扭矩扳手分子”的尺寸和极性能够控制二维纳米片的层间相互作用,从而进一步实现纳米片层间扭转角度分布的精确调控。在该工作中,由P-C_1_和P-C_2_诱导生成的Zr-BTB-P-C_1_和Zr-BTB-P-C_2_主要表现为旋转堆积,旋转角度分别为0°、12°、18°、24°及0°、6°、12°、15°、24°、30°;在延长侧链烷烃长度后,二维纳米片与“扭矩扳手分子”之间的疏水相互作用得到增强,由P-C_3_和P-C_5_诱导生成的Zr-BTB-P-C_3_和Zr-BTB-P-C_5_主要表现为规整堆积,规整堆积所占比例分别为64.8%和93.3%。此外,这项工作首次在单颗粒水平上发现,3种二甲苯异构体(邻、间、对)在旋转堆叠的Zr-BTB纳米片中表现出相似的脱附速率,而在规整堆叠的Zr-BTB纳米片中,二甲苯异构体的脱附顺序为间位、邻位、对位,表明规整堆叠的Zr-BTB纳米片具有良好的二甲苯异构体分离选择性。随后该工作又采用一系列酯类分子来诱导纳米片的层间堆积,验证了该策略的普适性,为开发更多新型2D-MOFs色谱固定相材料奠定了基础。

### 2.2 2D-COFs

COFs是一类由有机分子以共价键连接形成的新型多孔材料,其中2D-COFs的形成过程如下,有机分子仅在平面内通过共价键连接形成二维多边形片层,这些片层再通过层层堆叠构成多层框架结构材料,即2D-COFs。与2D-MOFs相比,2D-COFs通常具有更大的孔径,能够为尺寸较大的分析物提供更加合适的空间位阻效应,将其作为气相色谱固定相有利于大分子物质的分离。

2D-COFs往往倾向于形成小尺寸的多晶结构,而难以获得结构明确的大尺寸晶体^[30]^。Natraj等^[22]^通过溶剂热法,在常压条件下于5 min内实现了大尺寸2D-COFs单晶(由亚胺键连接)的成功制备。该工作采用苯胺和苯甲酸作为双调节剂,利用两种单体(1,3,6,8-四-(对氨基苯基)-芘(TAPPy)和对苯二甲醛(PDA))之间的聚合反应,成功制备出具有大尺寸片状的单晶TAPPy-PDA,并通过调控反应温度制备出了多晶TAPPy-PDA(图3);作为气相色谱固定相,单晶TAPPy-PDA具有良好的苯和环己烷分离能力,而多晶TAPPy-PDA固定相无法用于苯和环己烷的分离,表明2D-COFs的结晶性对色谱分离性能有着极其重要的影响。随后,Yusuf等^[31]^同样合成了上述单晶和多晶TAPPy-PDA,并将二者应用在反脉冲气相色谱(IGC)中,用于分离线性烷烃和一系列有机极性物质(乙腈、二氯甲烷、二乙醚、四氢呋喃和乙酸乙酯)。实验结果表明,相比于多晶TAPPy-PDA,单晶TAPPy-PDA能够实现更好的分离效果。为进一步解释单晶和多晶TAPPy-PDA的色谱分离差异,该研究还测试了单晶和多晶TAPPy-PDA的麦克雷诺兹常数,结果表明,单晶TAPPy-PDA未表现出极性特征,而多晶TAPPy-PDA却表现出微弱的极性特征。同时,通过计算二者的吸附自由能发现,单晶TAPPy-PDA具有电子供体特征,这可能归因于孔道中的亚胺氮原子;多晶TAPPy-PDA具有电子受体特征,可能来源于分析物和晶界上悬挂键或官能团之间的相互作用。由此可见,单晶和多晶TAPPy-PDA的极性和给电子能力均存在差异,因此二者在色谱分离中会展现出不同的分离性能。Ma等^[32]^通过在2D-COFs中引入羟基和三氟甲基,再利用原位生长法合成出了能够提供多重作用力的TpTFMB。将TpTFMB作为气相色谱固定相可以提供空间位阻效应、氢键、偶极-偶极相互作用、*π-π*相互作用等多重分离机制,从而实现对苯系物异构体(二甲苯异构体、氯甲苯异构体)、碳链异构体(丁基苯异构体、丁酸乙酯异构体)和顺-反异构体(1,3-二氯丙烯异构体)的基线分离,为用于异构体高效分离的二维COFs固定相设计提供了一种新策略。

**图 3:**
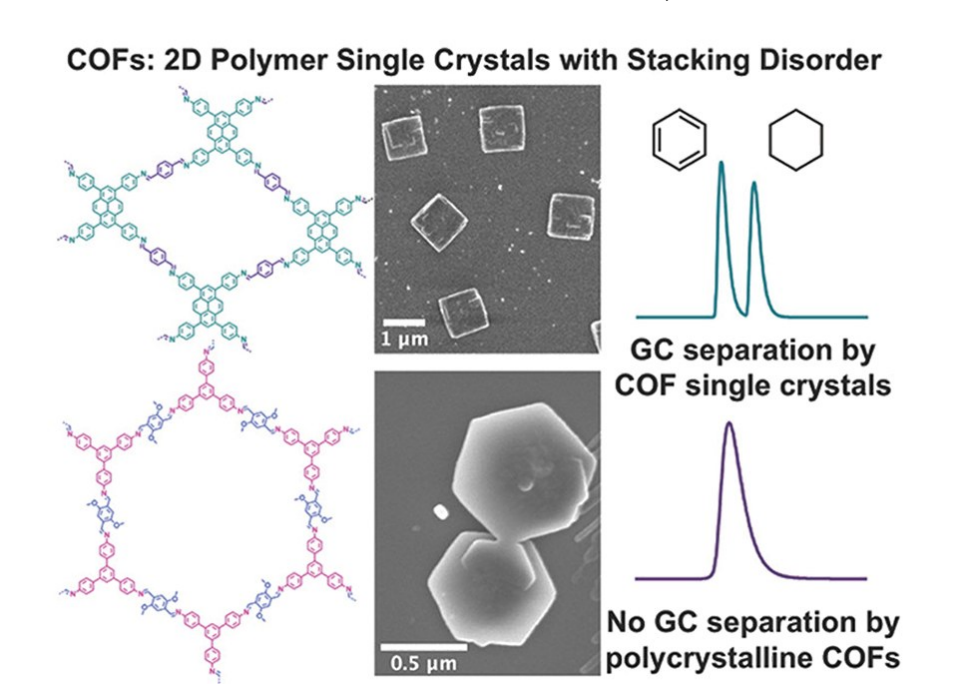
单晶和多晶TAPPy-PDA的结构、形貌及色谱分离性能^[22]^

Qian等^[33]^采用自下而上策略,通过在有机配体中引入手性中心,再利用双溶剂法直接合成出了手性COF。并且,通过调控合成时间及溶剂组分,得到了具有较好结晶性的手性层状2D-COF(CTpPa-1),之后通过原位生长法制备出CTpPa-1毛细管气相色谱柱。该毛细管气相色谱柱对手性异构体(1-苯基乙醇、1-苯丙醇、柠檬烯等)具有良好的分离性能,并且方法重现性较好。手性异构体的分离依赖于2D-COFs的手性微环境,而2D-COFs所提供的范德华力、氢键和*π-π*相互作用等也会影响手性色谱固定相的分离性能。此外,该工作还制备了另两种手性2D-COFs(CTpPa-2和CTpBD),证明了自下而上策略在手性2D-COFs合成中的实用性,同时扩展了2D-COFs在手性分离中的应用。

### 2.3 其他二维材料

除了多孔框架材料,二维材料还包括石墨烯、氮化碳石墨、氮化硼、过渡金属硫化物等;其中,石墨烯及其衍生材料(如氧化石墨烯(GO)、类石墨相氮化碳(g-C_3_N_4_)、六方氮化硼(h-BN))具有较大的比表面积,片层之间具有较强的*π-π*相互作用,为苯系物的高效分离奠定了基础。此外,石墨烯及其衍生材料通常具有优良的热稳定性和化学稳定性,有助于气相色谱柱重现性的提高^[34]^。Fan等^[35]^将石墨烯直接涂覆在熔融石英毛细管柱上,用于不同类型分析物的气相色谱分离。与商用HP-5MS色谱柱相比,石墨烯柱对极性分析物(醇类、羧酸、胺等)具有更好的分离效果和更快的分析速度。此外,石墨烯色谱柱对芳香族化合物也展现出了良好的分离效果,这主要依赖于石墨烯层间的强*π-π*相互作用。Li等^[12]^将石墨烯修饰在聚二甲基硅氧烷(PDMS)固定相中,修饰后的PDMS固定相对烷烃和醇类分析物的分离能力均有所提高。Han等^[11]^首先采用聚多巴胺对毛细管进行预处理,随后在毛细管内壁负载石墨烯固定相,与单独的聚多巴胺色谱柱和石墨烯色谱柱相比,所制备的色谱柱对烷烃异构体和丁醇异构体的分离性能更为优异;此外,芳香族化合物的保留时间普遍较长,该实验进一步验证了石墨烯材料在芳香族化合物分离中的重要作用。

2016年,Yang等^[36]^制备了石墨烯-ZIF8(G-ZIF8)复合材料,并将其作为气相色谱固定相用于烷烃异构体、几何顺反异构体和芳香异构体的分离。该固定相不仅能够利用石墨烯层间的*π-π*相互作用,还能充分发挥3D-MOFs的孔道选择性,进一步提高了色谱柱的分离性能。与单独的石墨烯气相色谱柱相比,G-ZIF8色谱柱对烷烃异构体展现出了更好的分离能力,同时对几何顺反异构体(二氯丙烯、十氢化萘)也展现出了良好的分离性能。此外,由于石墨烯与ZIF8的协同作用,G-ZIF8与分析物之间的*π-π*相互作用明显增强,对芳香异构体的分离性能也明显优于单独的石墨烯色谱柱和ZIF8色谱柱;并且与单独的石墨烯色谱柱相比,G-ZIF8色谱柱的热稳定性和重现性也更好。

继石墨烯之后,GO被开发出来,作为一种碳基材料,GO具有石墨烯的大部分优点。GO骨架上存在羟基、环氧基和羧基,与石墨烯相比,GO具有更强的亲水性和水溶性,其独特的表面性质为分析物的吸附提供了丰富的结合位点。2012年,Qu等^[37]^首次将GO作为气相色谱固定相,用于极性化合物的分离。该工作使用3-氨丙基甲基硅烷作为交联剂,将GO纳米片共价键合到毛细管柱内壁上,并在较低温度(30、50、90 ℃)下进行色谱分离。实验发现,在该GO气相色谱柱条件下,许多极性化合物(苯甲醚、苯酚、醇类)的色谱峰产生了明显的拖尾现象,表明GO与极性分析物之间存在极性相互作用,这可能归因于GO中的丰富官能团。与商业DB-1色谱柱相比,GO色谱柱对苯和1-丁醇具有更好的分离效果。除此之外,在引入其他固定相材料之前,先将GO涂层引入至毛细管内壁还可以消除硅醇基的非特异性相互作用。González-Álvarez等^[38]^将GO分散在二氯甲烷中并引入至毛细管内壁,随后将磷基离子液体固载至毛细管内壁上,制备出GO-离子液体气相色谱柱,用于农药分析物的分离。实验结果表明,GO的加入改善了色谱峰的展宽现象,分析其原因可能是GO增加了所负载离子液体的表面积,并在毛细管内壁上形成了更均匀的离子液体薄膜;此外,在GO加入前后,所有农药分析物的洗脱顺序保持不变,表明GO的加入不会干扰固定相对分析物的分离顺序。同时,该课题组的另一项研究^[39]^也证实了上述发现,该研究使用3-氨丙基甲基硅烷作为交联剂,将GO共价键合到毛细管柱内壁上,随后将磷基离子液体固载到毛细管内壁上,以提供更加均匀的离子液体涂层,从而使GO-离子液体复合柱的色谱分离性能得到了改善。

g-C_3_N_4_是一类近似石墨烯的平面片层材料,与石墨烯相比,其单层N含量更高,因此g-C_3_N_4_具有更高的化学稳定性。Zheng等^[17]^将纳米纤维状g-C_3_N_4_用于气相色谱固定相的制备,与块状g-C_3_N_4_相比,纳米纤维状g-C_3_N_4_具有海藻状的缠绕结构,能够提供更高的比表面积。将纳米纤维状g-C_3_N_4_色谱柱用于脂肪族和芳香族异构体的分离,结果表明,该色谱柱对脂肪族和芳香族异构体均展现出了良好的分离能力。

h-BN具有与石墨烯和g-C_3_N_4_类似的层状结构,不同的是,h-BN的硼原子和氮原子之间存在电负性差异,B-N键表现出明显的离子性,因此h-BN具有极高的化学稳定性和热稳定性。Xiong等^[40]^将h-BN作为气相色谱固定相,用于卤代烷烃、卤代苯及多环芳烃的分离,由于h-BN易与卤素键形成偶极-偶极相互作用,卤代烷烃和卤代苯在h-BN固定相中均表现出了较强的保留时间,并且二者均实现了有效分离。h-BN的热膨胀系数<0,在色谱分离过程中,随着柱温升高,h-BN逐渐呈现出褶皱状态(图4),这种褶皱可能使多环芳烃在h-BN固定相中的扩散路径发生改变。此外,该h-BN气相色谱固定相对芳香族和脂肪族异构体也表现出了良好的分离性能。

**图 4:**
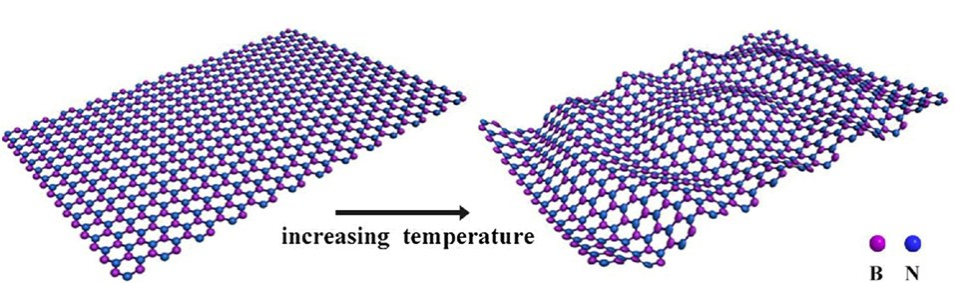
h-BN在不同柱温下的结构变化^[40]^

## 3 二维材料用于液相色谱固定相

### 3.1 2D-MOFs

2D-MOFs的孔隙结构简单,能够为分析物提供明确的扩散路径,因此2D-MOFs液相色谱固定相往往具有较小的传质阻力、较高的柱效率及良好的稳定性,是一种具有潜力的液相色谱固定相^[41,42]^。

Si等^[43]^首先利用原位生长法制备了3D-MOF-二氧化硅复合材料(MOF-808@silica)固定相,并将其应用在亲水作用色谱(HILIC)中,用于分离一系列极性化合物(磺胺类、生物碱、核苷、碱基、抗生素、氨基酸等);其中,MOF-808@silica固定相虽能够分离磺胺类、生物碱、抗生素、核苷和碱基等物质,但其仅能分离出5种氨基酸化合物,且色谱峰展宽较大,有明显拖尾现象,这主要是因为3D-MOF具有复杂的孔道结构,致使分析物的扩散路径混乱,动力学传输效率差。为改善上述色谱峰拖尾现象,Si等^[44]^将2D-MOF材料与二氧化硅结合,开发出2D-MOF-FDM-23@silica液相色谱固定相。与3D-MOF相比,2D-MOF具有更小的传质阻力和更高的柱效率,有利于分析物的传质与扩散。因此,2D-MOF-FDM-23@silica液相色谱固定相除了能够分离许多极性化合物(如磺胺类、生物碱和抗生素等),还能实现8种氨基酸化合物的高效分离。此外,2D-MOF-FDM-23@silica固定相在连续使用120 h后,仍具有较好的分离能力,说明该材料具有良好的稳定性。Si等^[23]^在2D-Zr-BTB上修饰聚多巴胺,随后通过双溶剂法制备出2D-Zr-BTB@PDA@silica液相色谱固定相。在制备过程中,利用两种极性不同的溶剂来协助聚多巴胺分子进入2D-Zr-BTB孔道,有效改善了2D-Zr-BTB@PDA颗粒在二氧化硅表面的团聚现象。在HILIC中,所制备的2D-Zr-BTB@PDA@silica液相色谱柱能够实现亲水化合物(磺胺类药物、核苷和核碱基、抗生素化合物等)的有效分离。在反相液相色谱中,聚多巴胺中的苯环结构增强了固定相与苯系物之间的*π-π*相互作用,从而实现了多环芳烃和烷基苯的分离。此外,Si等^[45]^还利用离子液体辅助合成了2D-Cu-BDC纳米片,并将其作为黏合剂,制备出2D-Cu-BDC与二氧化硅的复合材料(2D-Cu-BDC@silica)固定相。与不含离子液体的2D-MOF色谱柱和商业色谱柱相比,所制备的2D-Cu-BDC@silica色谱柱对磺胺类药物及生物碱等具有更好的分离效果。

### 3.2 2D-COFs

2D-COFs的结构中不存在金属中心,因此常见的生物大分子(如蛋白质)结构不易被破坏。当用作液相色谱固定相时,2D-COFs具有独特的优势,其层间存在强*π*-*π*共轭作用,具有高热稳定性和化学稳定性;并且,在液相色谱分析中,与2D-COFs固定相搭配使用的流动相选择性也更加广泛。

Wang等^[46]^使用一种新的cem拓扑结构制备出了3种2D-COFs,其中cem拓扑以一个吡咯分子为中心,并包含3个三角形孔道和两个矩形孔道。所制备的3种2D-COFs具有独特的孔道形状,并能够提供*π-π*相互作用和主客体作用,可以实现萘、苊、菲等10种多环芳烃的分离。Xie等^[47]^通过原位生长法在球形二氧化硅基底表面负载了2D-COFs(厚度约为98 nm),由于2D-COFs遮挡了二氧化硅自身的孔道,有效缩短了分析物的传质路径,实现了芳香族化合物(单取代苯、三联苯异构体等)的分离。

### 3.3 其他二维材料

Qu等^[48]^制备了一种涂有GO的毛细管液相色谱柱,与石墨烯毛细管液相色谱柱相比,GO毛细管液相色谱柱能够改善多环芳烃保留过强及色谱峰拖尾现象,并可以实现4种多环芳烃(甲苯、萘、2-甲基萘和苊)的高效分离。随后,Zhang等^[49]^分别制备了GO-二氧化硅复合材料(SiGO)和石墨烯-二氧化硅颗粒(SiG),并详细研究了二者的分离机制。该工作发现,SiG固定相对芳香化合物的分离主要依赖于*π*-*π*相互作用,而SiGO固定相的分离机制耦合了*π*-*π*相互作用、疏水相互作用和氢键。Borsatto等^[50]^将GO薄片负载到十八烷基(C18)功能化的氨基二氧化硅颗粒上,合成了3种尺寸(3、5、10 μm)的SiGO-C18ec颗粒,并制备成相应的复合SiGO-C18ec固定相;其中,颗粒尺寸为5 μm的SiGO-C18ec固定相展现出最高的柱容量和分离性能,并实现了8种具有不同极性和拓扑表面积分析物的良好分离。

## 4 总结与展望

本文总结了二维材料(2D-MOFs、2D-COFs及其他二维材料)在气相色谱和液相色谱固定相制备中的研究进展及应用。与传统的色谱固定相相比,二维材料色谱固定相的优势主要体现在以下3个方面:(1)二维材料的层状结构能够提供较大的比表面积,有助于目标分析物的吸附和分离,从而提高色谱分析的灵敏度和分离度;(2)二维材料的可设计性强,能够提供多重分离机制,以实现分析过程的优化和定制;(3)二维材料的孔隙结构简单,能够为分析物提供明确的扩散路径,使分析物具有更快的传质速度,有助于提高分离效率。然而,二维材料因其结构特异性也存在一些缺点,如适用性不够广泛、制备过程复杂、原料成本较高等问题,其中较高的制备成本限制了二维材料的大规模应用。因此,探索更加高效、简易、低成本的二维材料合成方法,或改进现有的二维材料合成方法非常必要。在二维多孔框架材料制备方面,配体分子轴向配位所带来的形貌改变是研究难点,在之后的研究中,需要开发出更加泛用的轴向生长抑制剂或寻找出能够抑制轴向生长的合成条件。此外,对已报道二维材料进行有效的后修饰或开发出二维材料与其他材料的复合材料也能够进一步扩展二维材料的应用潜力。目前,越来越多的二维材料色谱固定相被开发出来,用于提高色谱分离的灵敏度、通量以及选择性。然而,对于二维材料色谱固定相的研究主要集中在类石墨烯等传统二维材料上,对具备二维形貌的多孔框架材料研究还十分有限。目前,研究者对2D-MOFs和2D-COFs的认识往往来源于多孔框架材料,而对二者二维形貌本身的认知和研究缺乏系统性。此外,对二维材料分离机理的探究还不够深入,需针对二维材料的层状结构进行实验设计,并探究其分离机制,从而为二维材料色谱固定相的高效设计提供理论依据。随着新型二维材料的不断涌现,新的分离机制也会被不断揭示,二维材料在色谱分离领域将会发挥更大的作用。

## References

[1] Ainali N M, Kalaronis D, Kontogiannis A , et al. Sci Total Environ, 2021, 794: 148725 34323760 10.1016/j.scitotenv.2021.148725

[2] Young G M, Lurie I S. J Sep Sci, 2022, 45(1): 369 34535950 10.1002/jssc.202100513

[3] Lynch K B, Ren J, Beckner M A , et al. Anal Chim Acta, 2019, 1046: 48 30482303 10.1016/j.aca.2018.09.021

[4] Polyakova A, van Leeuwen S, Peters R. Anal Chim Acta, 2022, 1234: 340098 36328715 10.1016/j.aca.2022.340098

[5] Kowtharapu L P, Katari N K, Sandoval C A , et al. ACS Omega, 2022, 7(38): 34098 36188248 10.1021/acsomega.2c03387 PMC9520538

[6] Lubomirsky E, Khodabandeh A, Preis J , et al. Anal Chim Acta, 2021, 1151: 338244 33608083 10.1016/j.aca.2021.338244

[7] Liu B, Li H, Quan K J , et al. TrAC-Trends Anal Chem, 2023, 158: 116895

[8] Zhou Q L, Zhang P F, Jia L. J Sep Sci, 2011, 34(23): 3303 22083582 10.1002/jssc.201100567

[9] Yuan L M, Ren C X, Li L , et al. Anal Chem, 2006, 78(18): 6384 16970312 10.1021/ac060663k

[10] Wu Q, Hou X D, Zhang X F , et al. Talanta, 2021, 226: 122148 33676698 10.1016/j.talanta.2021.122148

[11] Han N, Qi M L, Ye M H, et al. RSC Adv, 2015, 5(90): 74040

[12] Li Y B, Zhang R Z, Wang T , et al. Talanta, 2016, 154: 99 27154654 10.1016/j.talanta.2016.03.037

[13] Yan M T, Long W W, Tao X P , et al. Chinese Journal of Chromatography, 2023, 41(10): 879 37875410 10.3724/SP.J.1123.2023.07029 PMC10599295

[14] Yang H, Tang W Q, Zeng C , et al. Chinese Journal of Chromatography, 2023, 41(10): 853 37875408 10.3724/SP.J.1123.2023.05002 PMC10599292

[15] Novoselov K S, Geim A K, Morozov S V , et al. Science, 2004, 306(5696): 666 15499015 10.1126/science.1102896

[16] Tao Z R, Wu J X, Zhao Y J , et al. Nat Commun, 2019, 10(1): 2911 31266966 10.1038/s41467-019-10971-x PMC6606621

[17] Zheng Y Z, Han Q, Qi M L , et al. J Chromatogr A, 2017, 1496: 133 28363415 10.1016/j.chroma.2017.03.060

[18] Yu R C, Yuan X. Prog Org Coat, 2024, 186: 107990

[19] Maiti B K, Maia L B, Moura J J G. J Inorg Biochem, 2022, 227: 111687 34953313 10.1016/j.jinorgbio.2021.111687

[20] Liu H L, Li Y N, Zi M , et al. Chinese Journal of Chromatography, 2023, 41(2): 187 36725715 10.3724/SP.J.1123.2022.06012 PMC9892976

[21] Liu J, Wu F, Gan L , et al. Chinese Journal of Chromatography, 2023, 41(10): 843 37875407 10.3724/SP.J.1123.2023.04021 PMC10598563

[22] Natraj A, Ji W, Xin J , et al. JACS, 2022, 144(43): 19813 10.1021/jacs.2c07166 36265086

[23] Si T T, Lu X F, Zhang H X , et al. Mikrochim Acta, 2021, 188(10): 360 34599383 10.1007/s00604-021-05023-5

[24] Mu Q Q, Zhu W, Li X , et al. Appl Catal B-Environ, 2020, 262: 118144

[25] Tang W Q, Zhao Y J, Xu M , et al. Angew Chem Int Ed Engl, 2021, 60(13): 6920 33480119 10.1002/anie.202014673

[26] Huang J M, Zhang X D, Huang J Y. Coordin Chem Rev, 2023, 494: 215333

[27] Zhang J, Chen Z L. J Chromatogr A, 2017, 1530: 1 29150064 10.1016/j.chroma.2017.10.065

[28] Lin Y T, Li Y L, Cao Y , et al. Chem Asian J, 2021, 16(21): 3281 34453404 10.1002/asia.202100884

[29] Tang W Q, Yi X, Guan H , et al. JACS, 2023, 145(49): 26580 10.1021/jacs.3c06731 38029332

[30] Liu H Y, Zhou Y, Guo J B , et al. JACS, 2023, 145(42): 23227 10.1021/jacs.3c07904 37843005

[31] Yusuf K, Natraj A, Li K , et al. Chem Mater, 2023, 35(4): 1691

[32] Ma T T, Yang C, Qian H L , et al. ACS Appl Mater Interfaces, 2023, 15(27): 32926 37367939 10.1021/acsami.3c05369

[33] Qian H L, Yang C X, Yan X P. Nat Commun, 2016, 7: 12104 27401541 10.1038/ncomms12104 PMC4945876

[34] Yang X H, Li C X, Qi M L , et al. RSC Advances, 2017, 7(51): 32126

[35] Fan J, Qi M L, Fu R N , et al. J Chromatogr A, 2015, 1399: 74 25937129 10.1016/j.chroma.2015.04.030

[36] Yang X H, Li C X, Qi M L , et al. J Chromatogr A, 2016, 1460: 173 27423773 10.1016/j.chroma.2016.07.029

[37] Qu Q S, Shen Y Q, Gu C H , et al. Anal Chim Acta, 2012, 757: 83 23206400 10.1016/j.aca.2012.10.032

[38] González-Álvarez J, Arias-Abrodo P, Puerto M , et al. RSC Advances, 2013, 3(44): 21377

[39] González-Álvarez J, Arias-Abrodo P, Puerto M , et al. New J Chem, 2015, 39(11): 8560

[40] Xiong X, Qi M L. J Chromatogr A, 2018, 1567: 191 30100014 10.1016/j.chroma.2018.07.008

[41] Firooz S K, Armstrong D W. Anal Chim Acta, 2022, 1234: 340208 36328716 10.1016/j.aca.2022.340208

[42] Jiang H, Yang K W, Zhao X X , et al. JACS, 2021, 143(1): 390

[43] Si T T, Ma J L, Lu X F , et al. ACS Applied Nano Materials, 2020, 3(1): 351

[44] Si T T, Liang X J, Lu X F , et al. Talanta, 2021, 222: 121603 33167271 10.1016/j.talanta.2020.121603

[45] Si T T, Lu X F, Zhang H X , et al. Chinese Chem Lett, 2022, 33(8): 3869

[46] Wang X, Han X, Cheng C , et al. JACS, 2022, 144(16): 7366 10.1021/jacs.2c01082 35418223

[47] Xie M C, Quan K J, Li H , et al. Chem Commun (Camb), 2023, 59(3): 314 36508301 10.1039/d2cc05650j

[48] Qu Q S, Gu C H, Hu X Y. Anal Chem, 2012, 84(20): 8880 22991893 10.1021/ac3023636

[49] Zhang X Q, Chen S, Han Q , et al. J Chromatogr A, 2013, 1307: 135 23932030 10.1016/j.chroma.2013.07.106

[50] Borsatto J V B, Maciel E V S, Lancas F M. J Chromatogr A, 2022, 1685: 463618 36345073 10.1016/j.chroma.2022.463618

